# Alzheimer’s disease and drug delivery across the blood–brain barrier: approaches and challenges

**DOI:** 10.1186/s40001-024-01915-3

**Published:** 2024-06-08

**Authors:** Iram Iqbal, Fatima Saqib, Zobia Mubarak, Muhammad Farhaj Latif, Muqeet Wahid, Bushra Nasir, Hamna Shahzad, Javad Sharifi-Rad, Mohammad S. Mubarak

**Affiliations:** 1 Department of Pharmacology, Faculty of Pharmacy, Bahauddin Zakariya University, Multan, 60800 Pakistan; 2 Punjab University College of Pharmacy, University of the Punjab, Lahore, Pakistan; 3 Primary and Secondary Healthcare Department, Govt of the Punjab, Lahore, Pakistan; 4 Department of Pharmaceutics, Faculty of Pharmacy, Bahauddin Zakariya University, Multan, Pakistan; 5 Department of Biochemistry, Bahauddin Zakariya University Multan, Multan, Pakistan; 6 Department of Biomedical Sciences, College of Medicine, Korea University, Seoul, Republic of Korea; 7 Department of Chemistry, The University of Jordan, Amman, 11942 Jordan

**Keywords:** Alzheimer’s disease, β-amyloid, Tau, Blood–brain barrier, Nanoparticles, Liposomes

## Abstract

Alzheimer's disease (AD) is a diverse disease with a complex pathophysiology. The presence of extracellular β-amyloid deposition as neuritic plaques and intracellular accumulation of hyper-phosphorylated tau as neurofibrillary tangles remain the core neuropathologic criteria for diagnosing Alzheimer's disease. Nonetheless, several recent basic discoveries have revealed significant pathogenic roles for other essential cellular and molecular processes. Previously, there were not so many disease-modifying medications (DMT) available as drug distribution through the blood–brain barrier (BBB) is difficult due to its nature, especially drugs of polypeptides nature and proteins. Recently FDA has approved lecanemab as DMT for its proven efficacy. It is also complicated to deliver drugs for diseases like epilepsy or any brain tumor due to the limitations of the BBB. After the advancements in the drug delivery system, different techniques are used to transport the medication across the BBB. Other methods are used, like enhancement of brain blood vessel fluidity by liposomes, infusion of hyperosmotic solutions, and local intracerebral implants, but these are invasive approaches. Non-invasive approaches include the formulation of nanoparticles and their coating with polymers. This review article emphasizes all the above-mentioned techniques, procedures, and challenges to transporting medicines across the BBB. It summarizes the most recent literature dealing with drug delivery across the BBB.

## Introduction

Alzheimer's disease (AD) is one of the most common and fatal neurodegenerative conditions in the world [1]. Over 50 million people worldwide are thought to have dementia [2]. More than 90% of all dementia cases on the globe are caused by Alzheimer’s disease (AD). AD frequently includes behavioral and psychological problems [3]. The illness often affects persons over the age of 60 [4, 5]. However recent investigations have revealed that the pathological alterations in AD start 15 years before the development of clinical symptoms and that by the time the diagnosis is confirmed, irreversible damage to the brain system has typically taken place [6]. Remembering loss and linguistic difficulties are signs of damage to the nerve cells involved in thinking, memory, and learning. The patient is unable to carry out regular tasks as a result, and gradually becomes bedridden, which ultimately results in death [7]. Moreover, epidemiological studies reveal that mild cognitive impairment (MCI) is normally regarded as a precursor stage of AD. Compared to the 1–2% conversion rate of AD in healthy aging, 10 to 15% of people with MCI develop AD each year [8]. Most of the cases—those classified as late-onset AD (LOAD)—occur after the age of 65, whereas those classified as early-onset AD (EOAD)—which account for less than 5% of all cases—occur before the age of 65 [7]. EOAD, which is thought to account for between 5 and 10% of cases of AD, has been reported more frequently recently [9].

Major risk factors for the condition include smoking, vascular problems, old age, head trauma, family history, inadequate physical activity, and environmental variables [10]. However, the diagnosis of EOAD is more challenging, frequently requires a longer time between beginning and diagnosis, and requires the use of further imaging and laboratory tests to find signs of pathological changes to support the AD diagnosis. Early detection of AD is, therefore, essential for the detection and treatment of the disease [11]. Furthermore, understanding the causes of illness initiation and progression is a requirement for the creation of drugs that treat the disease. In this respect, amyloid beta (Aβ) peptide-containing senile plaques, hyperphosphorylated arrangements of the microtubule-associated protein, i.e., tau protein in neurofibrillary tangles (NFTs), and neuroinflammation that results in neurodegeneration are the pathological characteristics of AD [12]. NFTs and Aβ plaques, which are produced by aberrant intracellular tau protein phosphorylation aggregation, are the two primary clinical hallmarks of AD [11].

On the other hand, the blood–brain barrier (BBB) consists of 3 cellular components: (1) endothelial cells, (2) astrocyte end-feet, and (3) pericytes (PCs). The bulk of blood-borne substances are prevented from entering the brain by the diffusion barrier that is generated by the tight junctions (TJs) between the cerebral endothelial cells [13]. A variety of neurologic diseases, such as stroke and neuroinflammatory disorders, are made more complicated by BBB dysfunction, which, for instance, impairs the TJ seal [13]. The costs of treating the condition are rising as the number of AD cases rises [14]. It is astounding how much the public health system and caregivers are affected. The disease disproportionately impacts women and frequently serves as the primary caregiver for loved ones who have AD. Approximately, 70% of those who care for AD patients and dementia are women [7], and research statistics show that depression affects 65% of caretakers [15]. There are currently few clinically effective disease-modifying therapies for AD, the most prevalent basis of dementia [16].

Disease-modifying medicines that may stop or decrease the progression of the disease are desperately needed, but sadly, none are present at this time. A never-ending string of failures of mid- to late-stage clinical trials has plagued the history of AD medication development. However, tremendous progress has been made lately in elucidating essential elements of the underlying pathobiology of AD. New therapy approaches are still being actively explored and tested even if the therapeutic pipeline has encountered difficulties, and several pharmaceutical companies have chosen to shutter their AD medication development sections. Recently Lecanemab is introduced for early stage AD. In a placebo-controlled study, it decreased amyloid markers in early Alzheimer's disease and produced a somewhat less severe loss on cognitive and function assessments. To ascertain lecanemab's safety and effectiveness in the early stages of AD, longer trials are necessary [17]. Lecanemab received its first approval for AD on 6 January 2023 in the USA under the Accelerated Approval Pathway. It is further undergoing regulatory review in the EU, Japan, and China, with clinical development proceeding in several other countries around the globe [18]. Based on the preceding discussion, this review outlines treatment options, obstacles, and opportunities on the road to developing disease-modifying medications. It also discusses recent developments in our understanding of the pathobiology of AD.

## Materials and methods

### Literature search and methodology

In the current review on drugs across the BBB in AD, relevant references published from 2000 to 2023 were acquired from various bibliographical databases such as Google Scholar, PubMed, Web of Science, Science Direct, and Scopus. In our search, we used keywords including “Blood–brain barrier”, “drug delivery system in neurodegenerative disorders”, “Alzheimer’s Disease”, and “preclinical and clinical studies”. The following criteria were used to choose the articles for this work: blood–brain barrier, challenges, approaches, and AD. Although we did not set any language limits for our search, we only included English-language articles for subsequent analysis. VOSviewer was used to analyze the published literature from 2010 to 2023 obtained from Pubmed. A network diagram based on keywords was constructed.

The mechanistic illustrated Figures were drawn in Microsoft PowerPoint 2019 and SMART (https://smart.servier.com//, accessed on 25 May 2023). The accompanying Figures were created using data from previously available literature.

## Alzheimer's disease

**Fig. 1:**
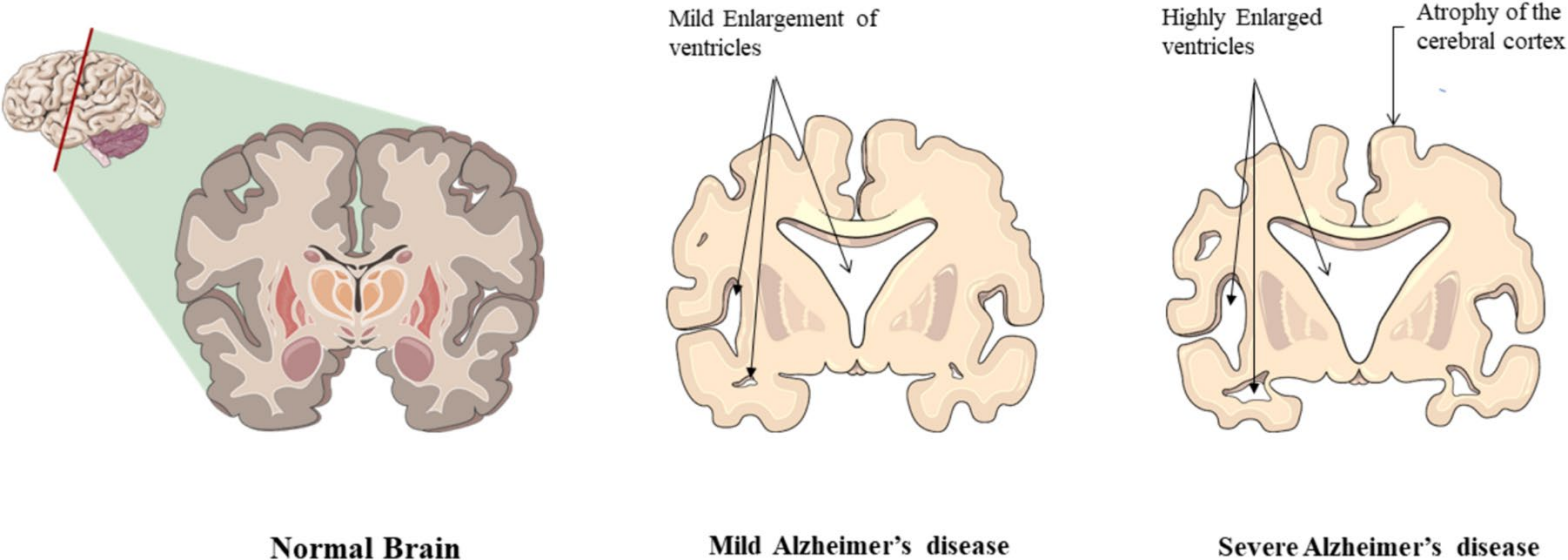
Alzheimer's disease development

Cognitive difficulties, memory loss, and behavioral abnormalities are the hallmarks of AD. The areas of the brain responsible for memory, learning, and higher executive functions are systematically destroyed by the disease. Although AD was first recognized more than a century ago, for a long time physiological changes that cause the disease were undiscovered [19]. In contrast, Aβ was originally discovered in 1984 and described as an endogenous neuropeptide that the central nervous system physiologically metabolizes. According to several noteworthy research, mild elevation of endogenous Aβ promotes long-term potentiation and causes neuronal hyperexcitability, while removal of this peptide causes synaptic malfunction and cognitive deficit [20]. The condition primarily affects the hippocampus, neocortex, and amygdala in the brain. Dementia from Alzheimer's disease affects more than 131.5 million individuals globally [21]. Para-hippocampal brain areas, which oversee the creation of new memories in the brain, are impacted by neuronal and synaptic damage during the disease's initial stage. Then, neuropathology spreads as the disease progresses, resulting in a 35% drop in total brain mass. The internal olfactory cortex, amygdala, and hippocampus are also atrophying, as are the frontal, temporal, and parietal cortex. There is also an increase in the breadth of the temporal horn of the lateral ventricle (Fig. 1) [22].

### Pathological hallmarks for AD

**Fig. 2:**
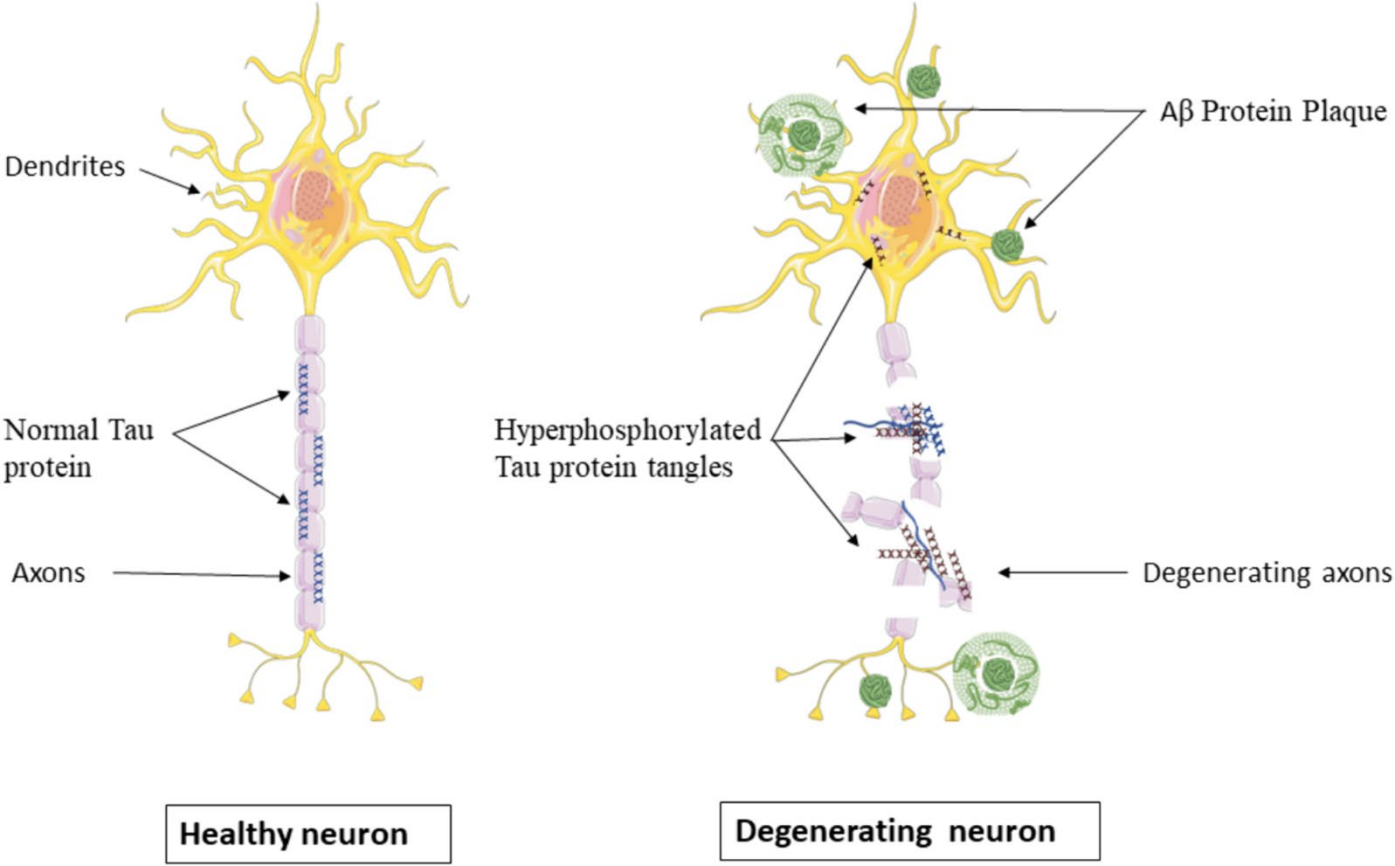
Pathological changes of neurons in AD

A healthy adult brain has around 100 billion neurons, each of which has long, branching extensions. These extensions make it possible for individual neurons to connect to other neurons. Information travels through these synapses, which are small chemical bursts emitted from one neuron and sensed by another. The synapses in the brain number in trillions. They enable quick signal transmission through the brain's neural circuits, forming the molecular basis for memories, feelings, thoughts, sensations, actions, and abilities [23]. Pathologically, AD is distinguished by hyperphosphorylated tau protein (known as tau tangles) in the form of NFTs inside neurons and beta-amyloid (referred to as beta-amyloid plaques) accumulation as diffused and neuritic plaques, in addition to neuronal and synaptic loss (Fig. 2) [24–26]. Both the development of intracellular neurofibrillary tangles containing tau and the buildup of extracellular plaques carrying amyloid protein defines the neuropathology of AD [5, 27].

#### Amyloid β protein

**Fig. 3:**
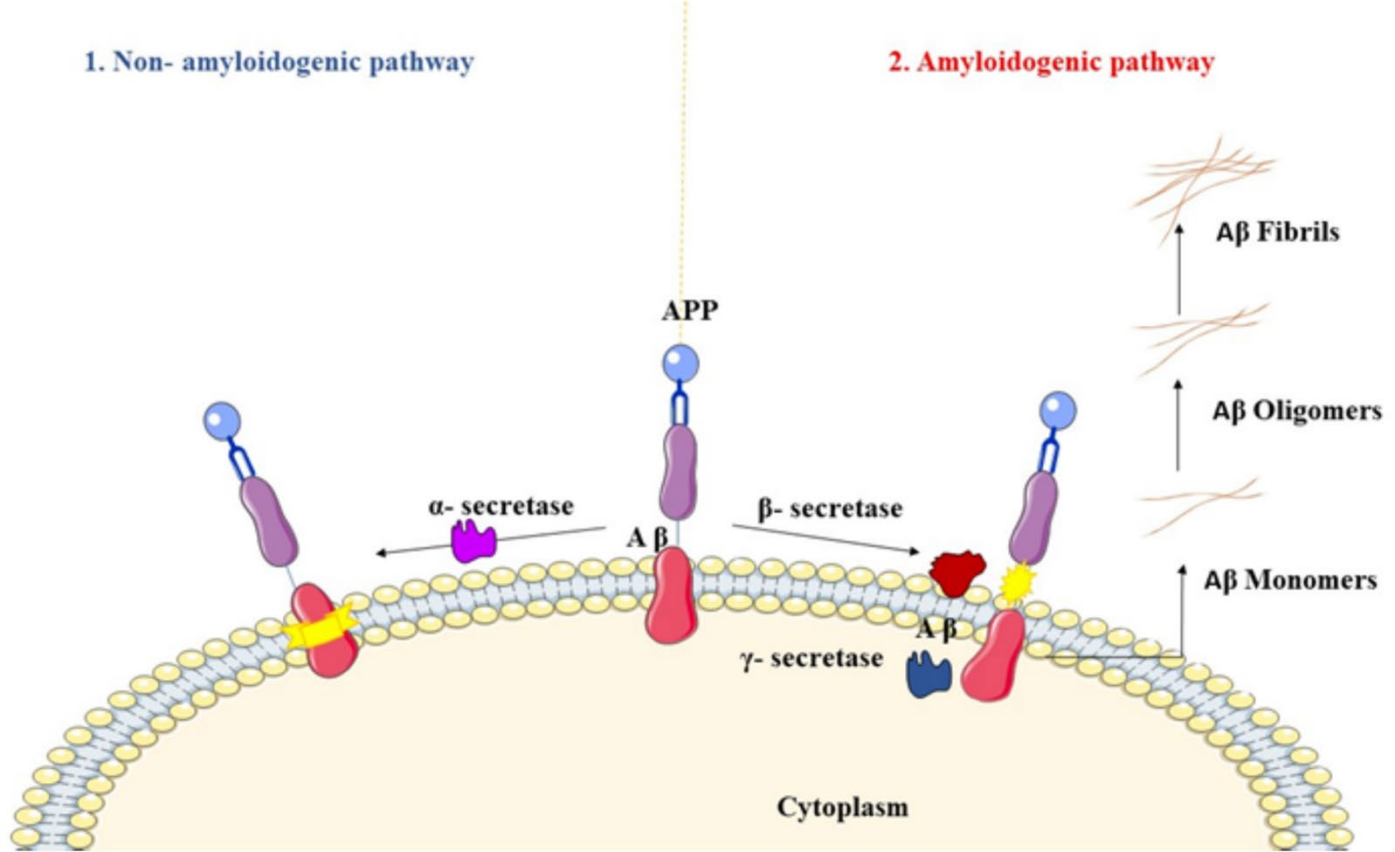
Processing of APP. (1) Non-amyloidogenic pathway: The enzyme α-secretase breaks down the amyloid precursor protein (APP) to produce extracellularly released, soluble APP. (left). (2) Amyloidogenic pathway: Inside the membrane, β-secretase cleaves APP in the first occurrence, followed by γ-secretase. (right). The proteolytic processing of APP opens the extracellular space through the amyloidogenic pathway, which produces amyloid-β, that is prone to self-aggregation and results in the development of cytotoxic type oligomers and insoluble Aβ fibrils

In 1984, the amyloid peptide was first sequenced and was later discovered to be the primary component of neuritic plaques [28]. The transmembrane amyloid precursor protein (APP) functions as a precursor of the A peptides, which make up amyloid plaques [29]. On human chromosome 21, there is a gene called APP that plays a role in synaptic plasticity, neuronal development, and the production and repair of synapses [30, 31]. An essential step in the development of AD is the proteolytic sequential cleavage of the amyloid protein by APP secretase. The non-amyloidogenic pathway and the amyloidogenic pathway are the two processing routes for APP: (1) In the middle of the amyloid chain, α-secretase cleaves APP, producing soluble APP α or shorter Aβ species when further cleaved by β-secretase [9, 31]. The term "non-amyloidogenic pathway" refers to this process (Fig. 3). In contrast, in the second pathway (2), the N- and C-terminal ends are sequentially cleaved by secretase, resulting in the production of soluble A peptides (monomers) [32]. In the presence of pathologic aggregates in the brain, soluble Aβ features conformational modifications that enable hydrogen bonding inter-molecules and produce very stable–sheet structures, resulting in brain dysfunction and neurodegeneration [23]. The second process, known as the amyloidogenic pathway, is responsible for the development of pathological Aβ [33]. Obstructing synaptic transmission between neurons, plaques, and more minor beta-amyloid accumulations known as oligomers may lead to the harm and decay of neurons (neurodegeneration).

#### Tau

Amyloid causes tau protein to become hyperphosphorylated and accumulate leading to neuronal and synaptic loss, and ultimately leading to clinical symptoms [34]. The tau protein filaments, which are known as NFT, abnormally form paired helical filaments (PHF) that can accumulate in neural-perikaryal cytoplasm, axons, and dendrites leading to loss of cytoskeletal structures including microtubules and tubulin-related proteins (Fig. 2). The brain of AD patients mainly has hyperphosphorylated tau protein in the form of NFTs. Its development can be used to interpret the morphological stages of NFTs, which include (1) one type of NFT called the “pre-tangle phase”, where phosphorylated tau proteins build up in the somatodendritic compartment without producing PHF; (2) mature NFTs, which are characterized by “tau protein filament aggregation” and somatic nucleus relocation to the periphery of the so-ma; and (3) the ghost NFTs stage or the extracellular tangles, that happens as a result of a neuronal loss brought on by an abundance of filamentous tau protein that is partially protease-resistant [35]. In the brain, the process of tau protein hyperphosphorylation causes the disintegration of microtubules and the development of insoluble neurofibrillary tangle clumps. Neurofibrillary tangles from Alzheimer's disease spread from internal brain regions to more distant areas, specifically from the transentorhinal cortex to the hippocampus and, ultimately, the neocortex [36]. These hallmarks result in neuronal malfunction, neurotoxicity, and inflammation, which disrupt memory and behavior and cause cognitive dysfunction [37].

### Stages of AD

AD clinical stages can be divided into three distinct categories: preclinical illness, MCI, and dementia caused by AD. Although the beginning and end points of the continuum are known—preclinical AD and severe Alzheimer's dementia—the length of time that people spend in each stage varies. In this regard, age, heredity, gender, and other factors have an impact on how long each phase of the continuum lasts [38]. Both cognitive deterioration and biomarker readings increase with time, but biomarker progression starts before symptoms appear [24].

#### Preclinical disease

At this stage, people show detectable brain biochemical changes that are the first symptoms of AD including raised levels of some biomarkers, but these patients have not yet shown symptoms like memory loss. For example, observable levels of aberrant beta-amyloid concentrations are present, and these are easily detected by “positron emission tomography (PET) scans” and “cerebrospinal fluid (CSF) studies”, as well as a diminished ability to metabolize glucose as detected by PET scans. The brain makes up for the early alterations of Alzheimer's, allowing people to carry on with their typical functions [39]. According to long-term observational research, those with cognitively normal brains are prone to developing dementia and MCI [40, 41]. It is crucial to remember that the existence of Aβ plaque is not always a prediction of dementia progress. For instance, some people die with beta-amyloid plaques but do not have memory or cognitive issues during life [23]. According to the most recent National Institute on Aging-Associated Alzheimer's disease (NIA-AA) research criteria, this pre-clinical AD stage corresponds to stages 1 and 2 [9].

#### MCI

Patients with MCI have minor issues with their memory and thinking capacity that may not be immediately obvious or cause interference with their aptitude to perform their daily living activities, in addition to the existence of biomarkers. These cognitive issues are not so obvious and may only be noticeable to close family and friends, as the brain is not able to repair the damage and loss of nerve cells caused by AD, which results in minor changes in thinking ability [20]. In contrast, prodromal AD includes people with MCI, which is defined as a deterioration in memory or another cognitive domain with no or minor impairment in daily living activities (ADL) [42, 43]. It fits the National Institute on Aging-Association Alzheimer's (NIA-AA) study criteria's stage 3 [9].

#### Dementia due to Alzheimer's disease

Dementia is categorized by a pattern of memory loss specified as intra-individual and reasoning impairment affecting at least two cognitive areas [44]. People who meet the requirements for a clinical diagnosis of dementia (NIA-AA stages 4, 5, 6) [9] are considered to have AD dementia or likely AD [45]. The phases of dementia are characterized as mild, moderate, and severe dementia, which represent the extent to which symptoms impair one's capacity to carry out daily activities [38].

### Alzheimer's disease risk factors

**Fig. 4:**
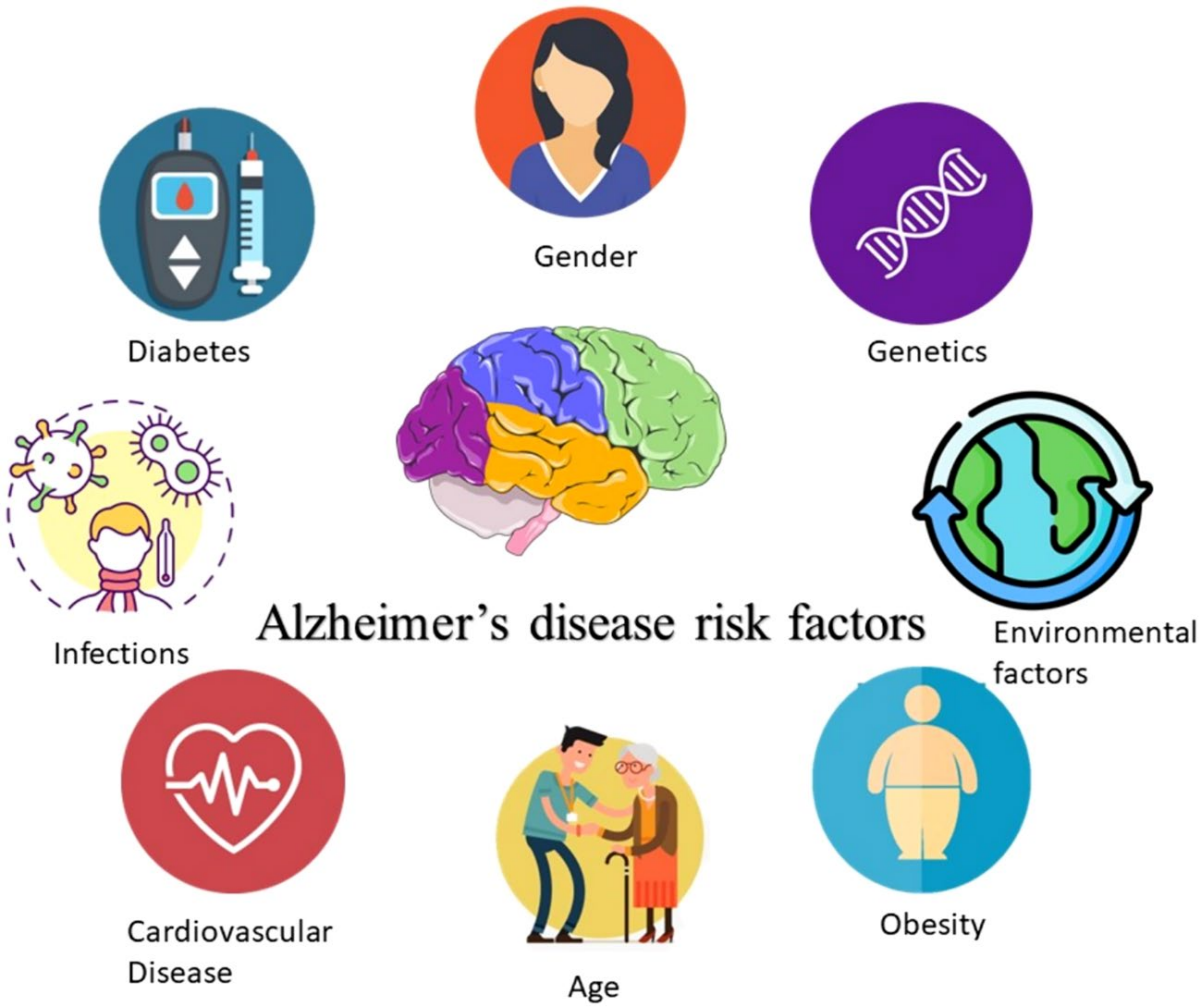
Factors increasing risk for Alzheimer's disease

Even though Aβ proteins are among the prominent mediators of AD and produce AD-related synapse loss and could lead to neuronal death, AD neuropathology comprises numerous additional risk factors that have been linked to an elevated risk of illness (Fig. 4) [46].

#### Aging

The leading risk factor for contracting illness is the phenomenon of aging. Numerous epidemiologic research studies have revealed that the leading cause of intellectual decline is aging [47]. The age of the patient where symptoms are noticed for the first time can be marked as a line to categorize the disease. Those people under the age of 65 are most likely to be affected by early-onset AD, whereas those in the age group 65 and above are victims of late-onset AD [11].

#### Genetics

Genetics is the 2nd most prevalent risk factor. Genetic factors account for 70% of the risk of contracting the illness [48]. Due to mutations in genes like the amyloid precursor protein (APP), presenilin-1 (PSEN-1), presenilin-2 (PSEN-2), and apolipoprotein E (ApoE), most instances of EOAD are hereditary by an autosomal-dominant form [49]. The APP gene has a total of 25 mutations that have been linked to AD and are responsible for the buildup of Aβ. In contrast, PSEN2 has rarer variants and < 40 mutations compared to the PSEN1 gene, which has more than 200 mutations linked to AD [50]. Where only 2 amino acid changes can result in some serious mutations, PSEN1 mutations frequently include just one. These PSEN1 gene variants enhance the Aβ42/Aβ40 ratio typically by decreasing the levels of generated Aβ40 [51]. However, mutations in the PSEN2 gene are sporadic and have little effect on the production of Aβ but are nonetheless linked to AD [50]. Three distinct alleles for the glycoprotein ApoE, which is abundantly produced in astrocytes, resulting in the formation of Ap-oE2, ApoE3, and ApoE4 isoforms. ApoE4 has been demonstrated to be essential for the development of Aβ as senile plaque and the primary risk factor for LOAD [52].

#### Gender

The anatomical and physiological differences between both genders have a significant impact on a variety of neuro-related disorders and diseases among men and women, including those of schizophrenia, multiple sclerosis, autism, and depression [53]. Men and women can both be affected by AD. However, the frequency of women affected by AD accounts for about 2/3 of AD cases. It suggests that women are more prone to develop AD as they age, have a stronger progression of mild cognitive impairment, and have clinical dementia that is more severe [46]. The physiological reasons behind these sex variations in the prevalence and severity of AD in women are not fully known [19]. Additionally, there are genetic variants, such as the ApoE4 allele, which cause significantly increased women's risk of AD compared to men's risk [54]. In this context, Buckley and colleagues conducted research on this topic in 2018, examining the connection between gender and cognitive impairment and amyloid beta load and ApoE genotype. Although there were no gender differences in the prevalence of ApoE4 or the load of A, females with higher amyloid beta burdens showed a quicker decline in cognition compared to males [55].

#### Environmental factors

In addition to aging, heredity, and sex differences, environmental risk factors like diet, metals, air pollution, and infections, which may promote reactive oxidative species leading to oxidative stress and inflammation, may increase the risk of developing AD [54].

#### Diseases

A few acquired variables increase the risk of getting AD. Li et al. [56] found a direct association between type 2 diabetes mellitus (TDM2) and an increased risk of developing AD [56]. Experiments using animals demonstrated that in addition to reducing insulin, insufficiency or resistance can also activate secretase, leading to its buildup in brain tissue [52]. CVDs are known as a noteworthy risk factor for AD. For example, stroke is responsible for cerebral tissue damage and can be linked to AD due to neuronal loss and acceleration of the degenerative process, thus affecting amyloid and tau pathology. Similarly, chronic cases of hypertension may end in cerebral edema atrophy and narrowing of the lumen of the artery walls, which limits cerebral circulation. All these changes contribute to the risk of AD. The link between AD and CVD risk factors can be used as a strategy to delay the onset of AD [57, 58].

### Diagnosis of dementia due to Alzheimer's disease

Notwithstanding the necessity for early and precise diagnosis, between 29 and 76% of dementia patients are thought to be undiagnosed [23]. A thorough cognitive and neurologic examination should serve as the foundation for the diagnosis, which should ideally include a history of the patient's close contacts regarding their cognitive status [23]. A comprehensive cognitive and neurologic examination must support the diagnosis, and ideally, it will also contain information regarding the patient's cognitive status from close contact [59]. Although it has been a component of the Medicare Annual Wellness Visit since 2011, just 47% of primary care physicians routinely test older patients for cognitive impairment, according to a recent Alzheimer's Association survey [23]. Direct observation of cognitive function should be part of the evaluation, which may also include a quick, standardized, and validated cognitive assessment method. Additional diagnostic tests should follow a positive cognitive screening test to confirm the diagnosis and identify the subtype of dementia [60]. Notably, the US Preventive Services Task Force panel concluded that there was insufficient data to support regular cognitive impairment testing in community-dwelling, asymptomatic people 65 and older [60].

#### Cognitive screening tests

The presence and course of dementia are measured using a variety of standardized mental state tests, such as the Mini-Mental State Examination (MMSE) and the Montreal Cognitive Assessment. Although these scales can discriminate clinically evident Alzheimer-type disease (CATD) from normal cognition with accuracy, they are less reliable when determining the difference between CATD and MCI and between moderate CATD and normal cognition [61].

#### Biomarkers

**Fig. 5:**
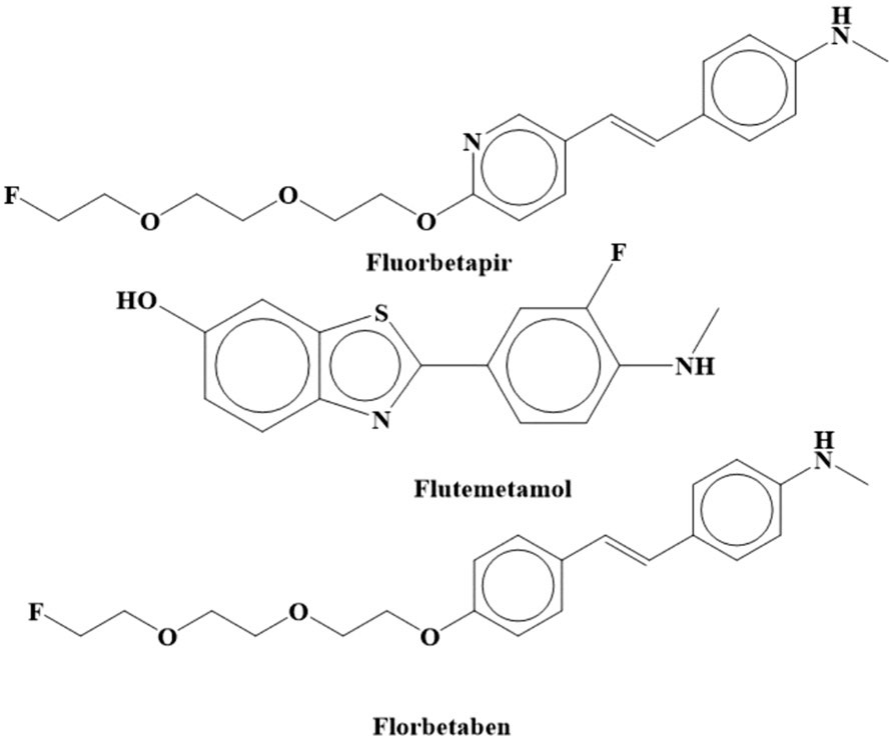
Chemical Structures of PET scanning agents

Since 15 to 30% of CATD patients do not match the postmortem diagnostic standards for AD, there is a severe unmet need for more precise diagnostic biomarkers [23]. Brain imaging and CSF biomarkers are two current diagnostic indicators that distinguish AD from other types of dementia. Florbetapir, flutemetamol, and florbetaben (Fig. 5) are “positron emission tomography (PET)” scanning agents that have FDA approval. If scan results can increase diagnostic inevitability and change the treatment strategy, the “Alzheimer's Association” and “Society of Nuclear Medicine and Molecular Imaging” encourage usage by dementia specialists assessing patients with cognitive impairment [62].

According to a recent systematic evaluation by the “Agency for Healthcare Research and Quality”, the Aβ-PET scan was highly sensitive and specific for the Aβ-pathological examination of AD and may improve categorization accuracy. The high expense of AD clinical trials is a result of the critical role biomarkers play in determining which individuals are eligible for treatment in clinical studies assessing disease-modifying medicines for AD. Each scan has an out-of-pocket expense of at least $3000. Medicare does not cover them unless they are carried out as part of a clinical trial evaluating if Aβ imaging improves patient outcomes or expands patient treatment options, despite commercial insurance coverage [14]. In the Imaging Dementia-Evidence for Amyloid Scanning (IDEAS) research, researchers investigated if PET scanning has an impact on how Medicare beneficiaries receive dementia care and how well it works [63]. More than 60% of patients in the study's initial phase had their treatment modified by doctors in response to scan results. While more than half of those whose AD was assumed to be unrelated had positive PET scans, more than a third of those whose AD was unrelated had negative ones.

Although no medication is effective in treating MCI, prescribing AD drugs increased from 40 to 82% in individuals with a positive PET scan (P < 0.001). Patients with dementia had an increase in prescriptions from 63 to 91% (P < 0.001). In patients with a negative scan, the prescription of AD medications fell a little [63]. It could not be long until the first MRI diagnostic tool for detecting tau pathology linked to AD becomes accessible. A PET scanning tracer called flortaucipir that binds to tau tangles is being considered for approval by the FDA. It had a sensitivity of 92–100% and a specificity of 52–92% for predicting tau pathology in a phase 3 postmortem validation investigation [64]. PET and CSF indicators for the presence of AD are intrusive, costly, time-consuming, risky, and not commonly used [14]. In this regard, clinically developed biomarkers may solve these constraints. According to validation studies, there is a blood test for plasma phosphorylated tau which predicted the levels of tau and Aβ pathologies recognized in AD across the clinical continuum and distinguished it from various other neurodegenerative disorders [65, 66].

## Blood–brain barrier (BBB)

An architectural and functional complex known as the neurovascular unit (NVU) is composed of neurons, glial cells (astrocytes, oligodendrocytes, microglia), and vascular cells (endothelium, pericytes, and vascular smooth muscle cells) [67]. The blood–brain barrier's (BBB) integrity is maintained by the cooperation of all of these elements, but especially the vascular cells. By maintaining the central nervous system (CNS) at home, the blood–brain barrier (BBB) promotes optimal synaptic and neuronal function. The intimate association between vascular structure and neurons in the central nervous system (CNS), which is vascularized, highlights the significance of neuro-angiogenesis for the CNS's functionality [68]. The selective border in this case is the BBB, which is made of multiple multicellular structures that prevent the flow of significant substances and immune cells from the circulatory system to the brain [69]. The BBB is a diffusion-based barrier that prevents most chemicals from entering the brain from the blood [13]. The BBB combines spatially different cell types to produce functional [68]. BBB's physiological characteristics also control how nutrients, chemicals, and medications are distributed from the blood to the brain and NVU [70]. The NVU is injured by midlife cardiovascular and metabolic risk factors (e.g., diabetes and hypertension), which initiates the pathologic disease cascade, according to the "two-hit" theory of AD’s etiology [71]. It has been postulated that this NVU damage results in disruption of the blood–brain barrier and decreased cerebral blood flow (CBF, first hit), which in turn causes decreased clearance of β-amyloid (Aβ) and the development of plaques containing Aβ [67]. AD and natural aging put a great deal of stress on the brain, especially the BBB, which changes the structure and, in turn, the functionality of the brain. In human and rat tissue, there have been reports of age-related cortical thinning, ventricular expansion, and increased BBB permeability. Evidence of faster BBB breakdown in the presence of AD disease has been demonstrated by the measurement of BBB permeability using K^trans^ in individuals with MCI or AD [2].

Additionally, intracellular metabolic activity can aid in the internal transportation of a variety of substances into the brain parenchyma. BBB restricts the passageway for medicines as well as shields neural tissue from hazardous substances and contaminants in the blood [72]. According to anatomy, the BBB is composed of polarized brain endothelial cells that are not fenestrated and are joined by tight junctions, i.e., claudin-5, occludin, zonula occludens-1, and adherent junctions such as VE-cadherin and β-catenin [73, 74]. The BBB is comparatively impermeable under physiological circumstances. Numerous chemical mediators are released under pathologic circumstances, increasing BBB permeability. These mediators of BBB opening, which are made by astrocytes and include glutamate, aspartate, taurine, ATP, endothelin-1, ATP, NO, MIP-2, tumor necrosis factor (TNF-), MIP2, and IL-, have all been investigated in both in vivo and in vitro research [13]. Bradykinin, 5HT, histamine, thrombin, UTP, UMP, substance P, quinolinic acid, platelet-activating factor, and free radicals are additional humoral factors that have been linked to increased BBB permeability [75, 76]. Moreover, P-glycoprotein, the human MDR1/ABCB1 gene's encoded product, is expressed in a variety of organs, including brain capillary endothelial cells, and functions as an efflux pump to limit the entry of foreign substances into the brain [77].

**Fig. 6:**
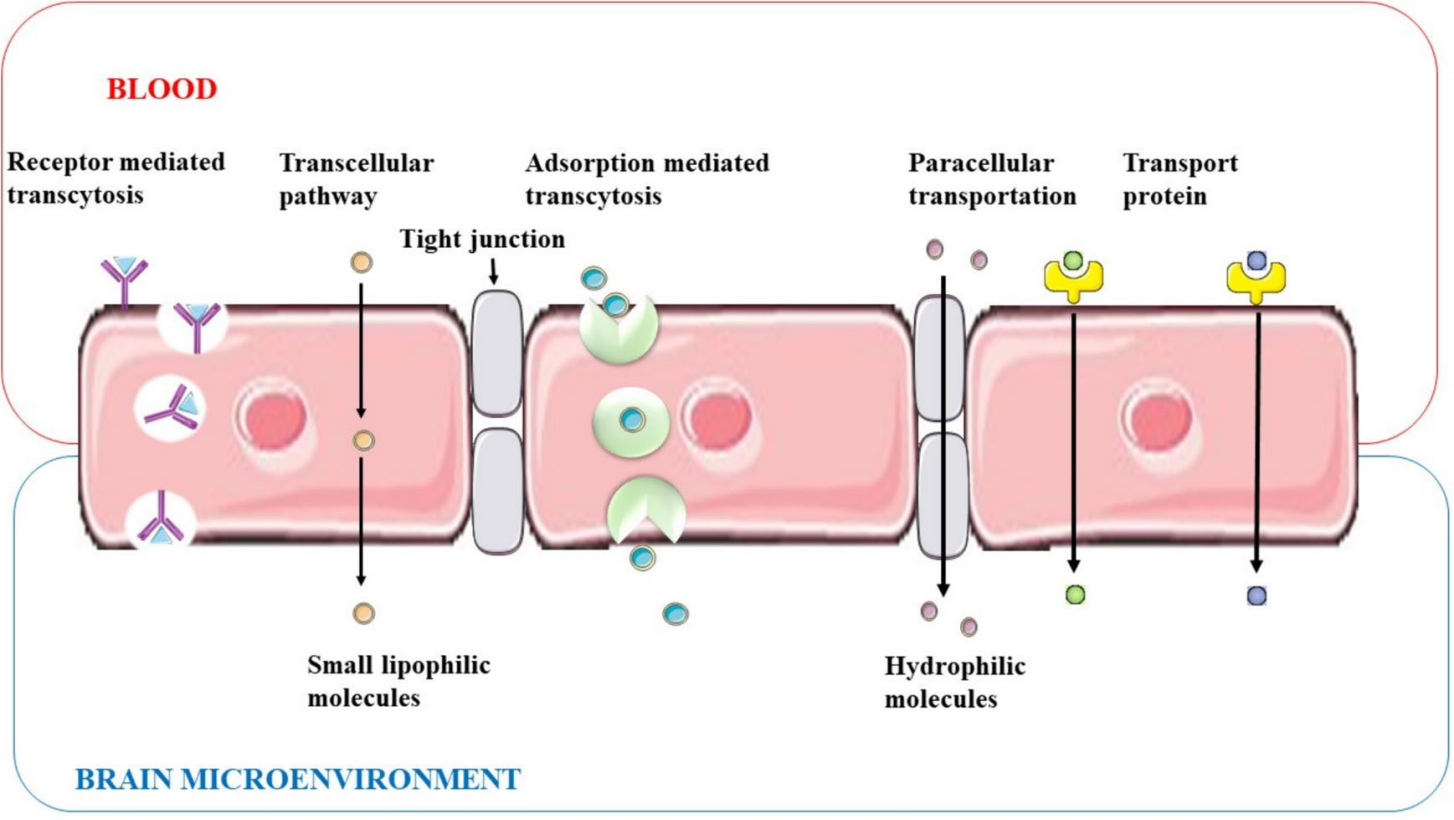
BBB-mediated solute transport from blood to brain

For the transportation of nutrients into the brain, the BBB has different incredibly selective processes. The BBB can be crossed by six different essential transport pathways for solute molecules (Fig. 6). The first one is “passive paracellular diffusion (PPD)”, which is the movement of materials through intercellular gaps between epithelial cells. Due to the presence of tight junctions, this route is almost nonexistent in the healthy BBB. The second method is “passive transcellular diffusion (PTD)”, in which molecules enter the intracellular space after passing through the bilayer cell membrane. The third type of “carrier-mediated endocytosis is solute carrier proteins (SCP)”, where solute molecules bind to membrane protein carriers, also from high to low concentration. Simple diffusion via a “receptor-mediated transcytosis (RMT)” comes in at number four, where the development of the transcytosis vesicle is induced by the binding of a serum protein to its transcytosis receptor on the apical side. The second method is called “adsorptive-mediated transcytosis (AMT)”, which works by engaging with negatively charged proteins on the surface of endothelial cells to carry drugs across the BBB. The last mechanism is tight junction modulation (TJM), which is difficult to detect in a healthy BBB and whose mode of action is not entirely known [78].

On the other hand, endothelial cells' tight connections can be momentarily broken, allowing for temporary permeability to systemic drug molecules and a physical bypass of the BBB. To cross the BBB, RMT has undergone substantial research into the delivery of antibody–drug conjugates [79–81], liposomes [82, 83], and nanoparticles [84, 85] to the CNS. Additionally, endogenous carrier-mediated transport mechanisms shown in the brain capillary endothelium may be used to build small molecule medications that can cross the BBB. Targeting the “endogenous RMT systems” shown inside the brain’s capillary endothelium, large molecules of medications such as recombinant technology-made proteins, peptides, and antisense radiopharmaceuticals will be transported through the BBB [86].

## Advancement in drug delivery methods in the brain targeted to cross BBB

**Fig. 7:**
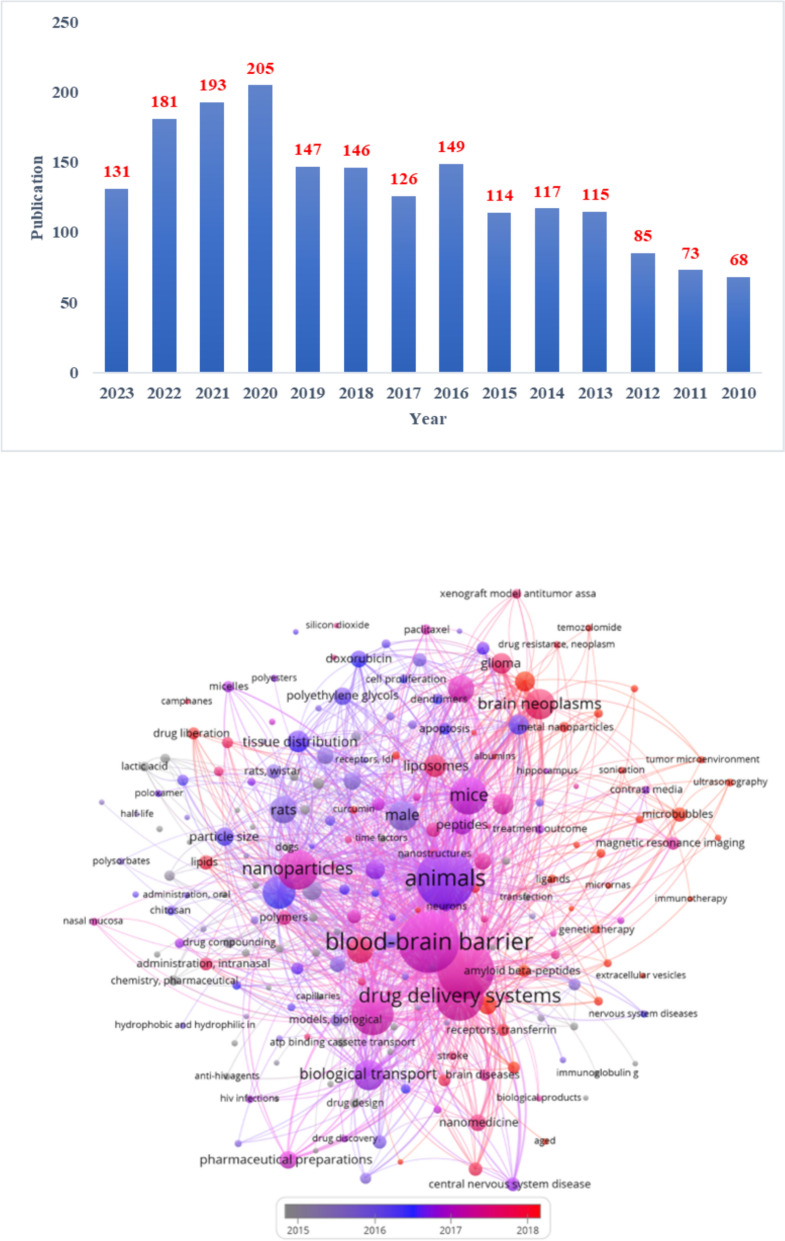
**A** The number of publications from 2010 to 2023. **B** The focused scientific keywords from 2010 to 2023

**Fig. 8:**
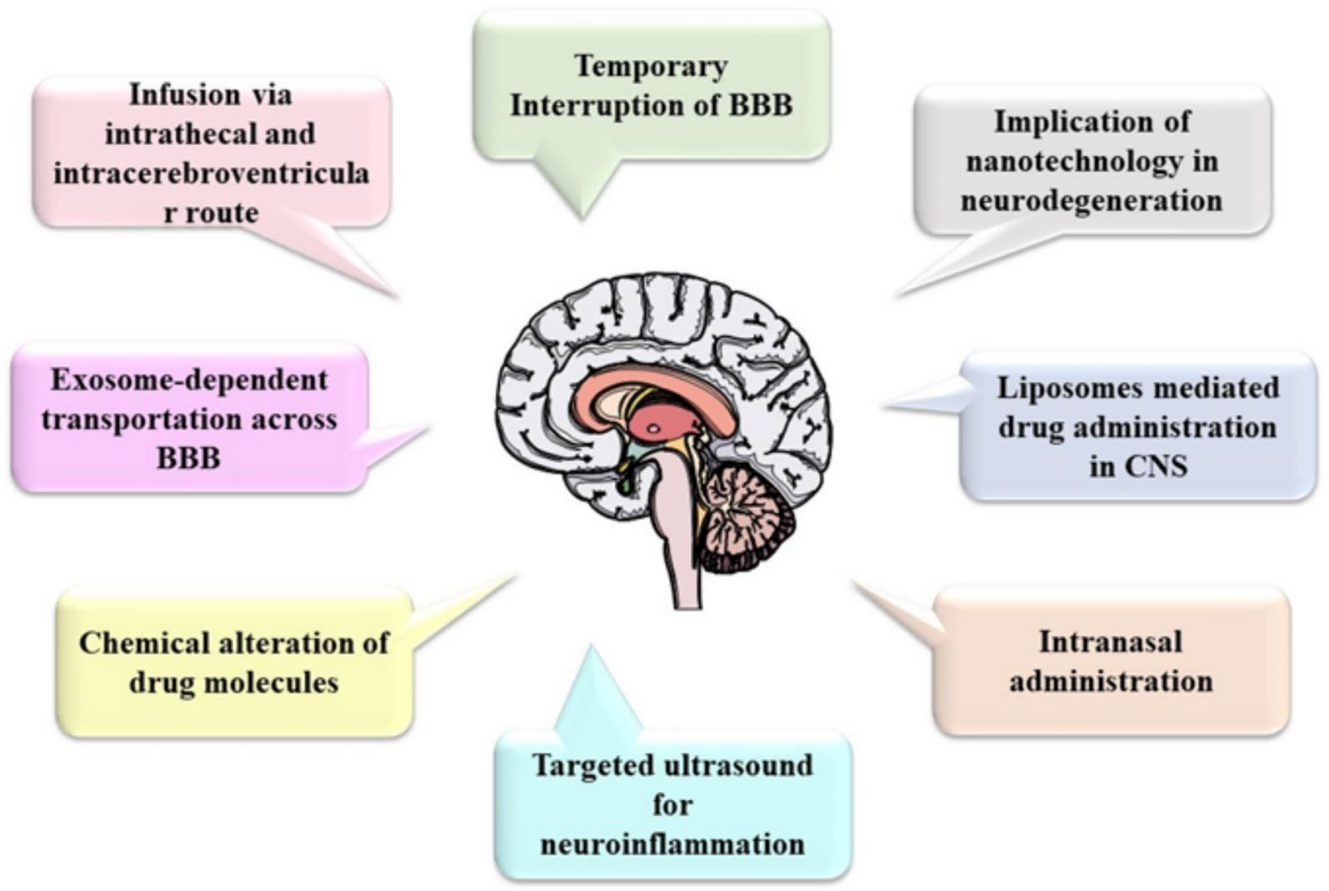
Brain-targeted drug delivery system in Alzheimer’s disease

We reviewed the scientific literature related to the subject from 2010 to 2023. During this period, 1640 articles were published. During our search, we used the keywords blood–brain barrier, and nanoparticles (Fig. 7A). Several advanced techniques are being followed for the enhancement of brain drug delivery in various neurological disorders, including AD (Fig. 8).

### Invasive technique

#### The temporary interruption of the BBB

This is an invasive technique in which the application of harmful light and compounds disrupts the integrity of the brain's endothelial cells, hence facilitating the entry of various substances into the cerebral tissue. These include rays of ultrasound and hyperosmotic preparations, including polysorbate 80, ethanol, mannitol, glycerol, metals, and dimethyl sulfoxide [87]. This method has notable limitations; it lacks patient-friendliness and carries the potential to harm the structural integrity and neurobiological performance of the BBB. Consequently, there is a risk of accumulating undesirable blood constituents and harmful agents including foreign substances, neurotoxicants, and xenobiotics, which can lead to CNS injury [88]. Electroporation (EP) is a novel method that has been suggested to induce temporary and localized disruption of the BBB. During the process of EP, cells or tissues are subjected to pulsed electrical fields (PEFs), which leads to the disruption of the electrical potential across the cell membrane [89]. Alterations in the electrical potential instigate the creation of nanoscale aqueous pores within the membrane, hence augmenting the permeability of the lipid bilayer. The process of cellular membrane resealing is generally referred to as reversible EP. Conversely, if the application of PEFs results in cellular damage, it is known as a non-reversible EP [90]. Previous research demonstrated the potential for inducing transient BBB deterioration in rodents using contrast-enhanced T1-weighted magnetic resonance imaging (MRI) following EP therapies [91].

#### *Infusion *via* intrathecal and intracerebroventricular route*

Another invasive technique involves the administration of therapeutic proteins through intraventricular infusion (CSF), involving direct injection or infusion into the cerebrospinal fluid [81]. The benefits of these methodologies compared to systemic enzyme replacement therapy (ERT) lie in their ability to facilitate the transportation of a larger quantity of enzymes to the brain. Accordingly, they obviate the need for administering excessively high doses of therapeutic drugs. In addition, these proposed solutions effectively tackle the challenges related to the limited duration of pharmaceutical effectiveness within the body while also mitigating the risks associated with widespread distribution and potential toxicity. To administer intrathecal drugs (IDDD), medical professionals may employ either a device for intrathecal implantation or lumbar puncture [92].

According to results obtained from laboratory animal studies of Mucopolysaccharidosis (MPS), administration of ERT through intrathecal injection allows the distribution of the recombinant enzyme throughout CNS. This distribution facilitates the enzyme's penetration into brain tissue, initiating the process of enhancing the elimination of accumulated substances within the lysosomes [93]. Human clinical trials have commenced to evaluate the protection and acceptability of recombinant human heparan-N-sulfatase (rhHNS) given through the intrathecal route in patients diagnosed with MPS IIIA. These trials aim to build upon the promising outcomes observed in animal studies, which demonstrated that direct recurrent infusion of a deficient enzyme through injection into the cerebrospinal fluid effectively treated pathological brain alterations in dogs and rodents (NCT01155778 and NCT01299727) [94, 95]. Likewise, an investigation of the toxicity of idursulfase, designed explicitly for intrathecal administration (idursulfase-IT), on patients with MPS II was conducted using IDDD [96]. Results of these experiments catalyze further investigations, although the practical use of these processes in a clinical setting is considered challenging due to the limited duration of the enzymes' activity. The administration of many doses, accompanied by an escalated likelihood of unfavorable outcomes, is necessary to enhance efficacy and heighten the potential for achieving positive clinical outcomes.

### Non-invasive technique

Non-invasive strategies primarily involve pharmacological interventions that can modify pharmaceutical agents to facilitate their transport across the BBB.

#### Exosome-dependent transportation across BBB

Exosomes are endogenous membranous vesicles that are ubiquitously present in many cellular compartments throughout the human body. During the previous two decades, significant advancements have been made in comprehending exosomes, encompassing their functionality, mechanisms, and constituents. Extracellular vesicles, which are naturally occurring nanoparticles capable of facilitating intercellular communication, are being recognized as potential pioneers in the field of medicine. They hold promise for various implications, including preventive measures, therapeutic interventions, and advancements in pharmacology and gene therapy [97]. To accomplish this purpose, a variety of nano- and micro-scale structures, including extracellular vesicles (EVs), are utilized [98]. Based on prevalent scientific hypotheses, it is postulated that EVs are generated by the mechanisms involving the plasma membrane and endosomal-linked processes. Exosomes, as previously mentioned, are important categories of EVs, originating from endosomes and typically measuring 50–100 nm in diameter [99]. The important site within the BBB responsible for regulating exosome transit is the endothelial cells (EC). When circulatory exosomes interact with BBB ECs, several common mechanisms of EV uptake are employed. These mechanisms encompass the augmented entry of exosomes from the circulatory system into the cerebral tissue, as well as phagocytosis, plasma membrane fusion endocytosis, and micropinocytosis [100].

Under normal conditions, it is widely acknowledged that exosomes possess the capability to traverse the BBB. It is essential to recognize that the occurrence of inflammatory conditions has the potential to alter the endothelial lining of the brain, leading to heightened exosome transfer and permeability. One issue that substantiates this concept is the swift exosome translocation from the CSF into the blood after the initiation of central nervous system (CNS) inflammatory changes [101]. Several studies have indicated that a significant proportion of exosomes that are administered systemically are rapidly sequestered in the spleen, liver, and lungs. The phenomenon can be ascribed to the existence of intricate networks of capillaries and specific populations of immune cells that possess receptors capable of phagocytosis [102]. Multiple experiments have demonstrated that unchanged exosomes can readily distribute in body fluids by unhindered dispersion, lacking any specific targeting capabilities. Consequently, surface modification strategies can be employed to modify the targeting capabilities of exosomes [103]. Exosomes can be considered effective vehicles for delivering medication to the parenchyma of the brain. To optimize the interaction between exosomes and endothelial cells (ECs) at the BBB and improve exosome passage, researchers could potentially enhance the effectiveness of the delivery of medication to the abluminal side of the BBB. The attainment of this objective necessitates a more profound understanding of the fundamental mechanisms implicated in the transportation of exosomes across the BBB, along with the accompanying obstacles.

#### Chemical alteration of drug molecules

The primary limitation that must be considered for chronic CNS conditions, including both neurological disorders and tumor formation, is the requirement of a transvascular approach before systemic administration. Due to the limited efficacy of small-molecule therapeutics in addressing numerous severe neurological illnesses, utilization of big molecules, therapeutic peptides, inhibitors, or other drugs becomes imperative [87]. Consequently, the transportation of medication to the brain necessitates the utilization of techniques such as nanoparticles that possess the ability to transport pharmaceutical substances across the BBB. The BBB functions to selectively restrict the passage of hydrophilic medicines and most molecules, save for specific lipophilic molecules of very small size (about 300 Da) that can infiltrate by diffusion [104].

Lipidation of a medication has been explored as a means of facilitating its transport to the CNS. Nevertheless, this strategy has encountered restricted achievements due to the trade-off between enhanced lipophilicity and increased biodistribution. Most transporters exhibit selectivity, thus necessitating the drug to mimic the natural ligand in this technique [105]. The Maillard process, along with glycosylation or glycation, has the potential to enhance the transportation of medicine and peptides to the brain while also improving their biological stability.

#### Targeted ultrasound for neuroinflammation

In recent years, the use of ultrasonography has gained significant popularity to facilitate the transportation of drugs through the BBB. Microbubble-enhanced diagnostic ultrasonography (MEUS) is a non-surgical technique used to expedite the passage of drug molecules via the blood–brain tumor barrier (BBTB) by augmenting its permeability in patients with glioma. The primary proteins found in tight junctions (TJs) within the BBB consist of junctional adhesion molecules (JAMs), occluding, and claudins. The use of ultrasonic irradiation in conjunction with microbubbles has the potential to inhibit the expression of tight junction (TJ) proteins [106], initiating BBB permeability within a limited timeframe while minimizing harm to healthy brain tissue [107]. Additionally, Ningaraj et al. observed that MEUS led to an upregulation of calcium-activated potassium (KCa) channels in glioma cells. This upregulation facilitated pinocytosis and subsequently increased BBTB permeability. The BBB, together with the BBTB, continues to provide challenges in the effective delivery of medication to tumors of CNS. The BBB serves to hinder the potential movement of immunobiological agents and various drugs. Consequently, research efforts have focused on methods to temporarily enhance its permeability. This enhancement aims to facilitate the delivery of immunotherapeutic drugs intended to combat beta-amyloid plaques found in neurodegenerative conditions like Alzheimer’s disease. Studies utilizing FUS (focused ultrasound) with contrast microbubbles have been conducted in rats, rabbits, and monkeys to explore this avenue [108]. The use of focused ultrasound (FUS) in conjunction with microbubbles has been observed to augment the permeability of the BBTB in a targeted manner, while concurrently inducing disruption of the BBB in the adjacent tissue [109]. Non-human primates were subjected to the administration of focused ultrasound (FUS) at different sonic pressures to investigate the physiological alterations in the brain resulting from the opening of the BBB induced by FUS [110].

#### Intranasal administration

The delivery of medications directly to the brain using intranasal (IN) injection has several advantages in the management of neurodegenerative diseases. The BBB imposes limitations on the efficacy of novel therapeutics intended for the treatment of memory loss and neurodegeneration, as it inhibits their penetration into the brain based on factors including drug size and charge [111]. Intranasal administration can traverse the endothelial lining of the brain and presents a non-invasive alternative to invasive methods of medicine delivery by directly introducing medications into the brain through the nasal cavity. Numerous neuroleptics, including those with the ability to traverse the BBB upon systemic administration, exhibit advantages when delivered through non-invasive IN administration. This route of administration specifically directs CNS therapy, thereby diminishing exposure to systemic circulation and minimizing potential toxicity. The process of delivering therapeutic substances to the CNS does not typically require modifications to existing CNS therapies. Furthermore, the administration of therapeutic agents to the CNS is expeditious, normally taking just a matter of minutes [112].

In 1989, Frey introduced the IN-delivery approach to transport neurotrophic factors, including fibroblast and nerve growth factor (NGF) into the brain. Intranasally administered therapeutics get access to the CNS by utilizing the trigeminal and olfactory cranial pathways. The innervation of the nasal cavity is derived from both the olfactory and trigeminal nerves, establishing a direct connection to the central pathways. Previously, it was widely accepted that the olfactory pathway was responsible for the direct transportation of drugs from the nasal cavity to the brain [87]. Recognition of the trigeminal pathway's role in the transportation of medication from the peripheral nervous system to the CNS, specifically to the caudal regions of the brain and spinal cord, has emerged as a new development. The administration of neurotrophins through a non-invasive approach has emerged as a potential therapeutic option for targeting the CNS and addressing neurodegenerative conditions, as supported by accumulating evidence. Research findings showed that the delivery of NGF results in a reduction of neurodegeneration and an improvement in cognition in a rodent model that mimics AD [113]. Additionally, it was shown that the administration of insulin-like growth factor-I (IGF-I) resulted in a reduction in neurological destruction and improvements in CNS deficits in a murine model of stroke [114].

#### Liposomes-mediated drug administration in CNS

Extensive research has been conducted on liposomes about their potential application in drug administration and invasive bioimaging for diagnosing and strategic treatment of glioma, AD, and Parkinson's disease. This interest stems from the distinctive physicochemical characteristics exhibited by liposomes [115]. In this respect, liposomes have unique physicochemical qualities that enable them to encapsulate therapeutic compounds with hydrophilic, lipophilic, and hydrophobic characteristics. Hydrophilic chemicals are frequently positioned near the boundary between the lipid bi-layer and the surrounding aqueous phase, or they might be enclosed within the watery core of liposomes. Medications that are lipophilic or hydrophobic are predominantly sequestered within the hydrophobic core of lipid bilayers in liposomes. The utilization of cationic lipids also allows the absorption of polyanions such as DNA and RNA [95].

Additionally, these materials demonstrate robust biocompatibility and biodegradability, along with negligible toxicity, targeted drug delivery capabilities, and controlled release of drugs. The liposomal membrane has the potential for modification through the incorporation of macromolecules, such as polymers, polysaccharides, peptides, antibodies, or aptamers. This modification aims to improve the liposomes' circulation within the bloodstream and permits efficient targeted delivery to the brain [95]. Unfortunately, the utilization of liposomes for the targeted administration of drugs to the CNS is not presently employed in clinical settings [116]. However, it should be noted that a number of these substances are either approved for clinical usage or are undergoing clinical trials [117]. The practicality and effectiveness of enhancing medicine bioavailability in the brain through the intranasal administration of rivastigmine [118] or galantamine [119] liposomes have already been shown.

## Implication of nanotechnology in neurodegeneration

### Polymer-based nanoparticles

**Fig. 9:**
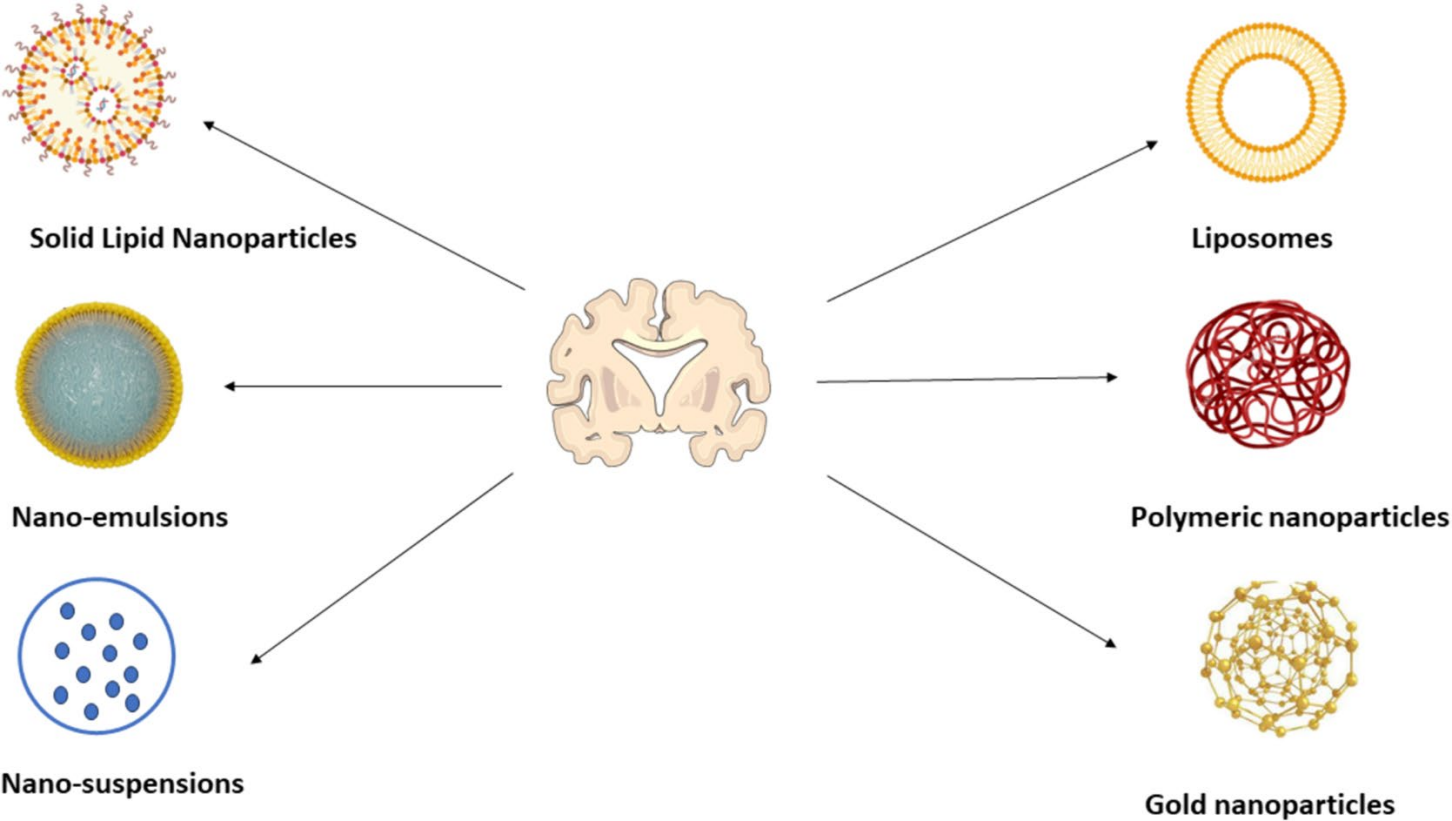
Nanoformulations for improved drug delivery across BBB

Nanoparticles (NPs) are characterized as colloidal dispersion or solid entities with dimensions spanning from 1 to 1000 nm. The arrangement of a nano-system is determined by its constituents. Nanocapsules have compartments that consist of either an oily core or an aqueous core, which are enveloped by a thin polymeric membrane. On the other hand, nanospheres exhibit a matrix-like organization of the polymeric chains [120]. The transportation of drugs across the BBB to reach the brain presents a notable potential benefit compared to existing approaches, as it avoids compromising the integrity of the BBB (Fig. 9).

The transportation mode of NPs across BBB can be elucidated by the enhanced accumulation of NPs within the cerebral blood capillaries, along with the adsorption of NPs onto the endothelial walls of these capillaries. These activities mentioned above result in an elevated concentration gradient, thereby augmenting the transportation through the endothelial cell layer and ultimately facilitating the delivery to the brain [121]. NPs have the potential to cause adverse effects on the vasculature of the brain, resulting in a restricted permeability of the endothelial cells within the brain. The application of a surfactant to solubilize the lipids present in the endothelial cell membrane has the potential to augment the permeability of drugs across the BBB. The NPs can traverse the BBB by exploiting the permeability of the tight junctions, which are selectively open gaps located between consecutive endothelial cells of the cerebral blood arteries [122]. The process of endocytosis by endothelial cells, subsequently leading to the intracellular release of medicines, enables efficient delivery to the brain. Transcytosis is a method that can additionally allow transportation via the endothelial cell layer [123].

Nasal administration of NPs can also be employed to enhance absorption and facilitate targeted distribution to the brain [124]. In this case, additional technological approaches involve the utilization of polyethylene glycol (PEG) polymers or antibodies to improve the nasal absorption of NPs [125]. The application of mucoadhesive polymers for surface modification of nanoparticles enhances the duration of nanoparticle retention when administered through the nasal route [126]. Wilson and colleagues conducted a study in which they synthesized NPs loaded with tacrine using emulsion polymerization. These NPs were coated with polysorbate 80 and made from poly (n-butyl cyanoacrylate). The levels of tacrine in the pulmonary and renal tissues did not exhibit statistical significance compared to the control groups. These researchers proposed a delivery strategy for the coated polysorbate 80 NPs to the brain by leveraging the interaction between the polysorbate 80 coating and the endothelial cells present in the brain microvessels [127]. In a separate investigation, Wilson et al. conducted research on the development of poly (n-butyl cyanoacrylate) NPs that were coated with polysorbate 80. The purpose of the study was to explore the potential of these NPs for delivering rivastigmine specifically to the brain, intending to treat AD [128]. The experimental procedure involved the administration of NPs through injection in mice to conduct animal investigations. The concentration of tacrine in the brain was found to be around 170 ng/mL when the coated NPs were administered. This observed outcome was statistically significant (P < 0.001) compared to the administration of uncoated NPs or the free medication. According to the authors, the proposed method for facilitating the transportation of coated polysorbate 80 NPs to the brain involves the interaction between the polysorbate 80 coating and the endothelial cells present in the microvessels of the brain [128]. The significance of the poly-sorbate 80 coating in facilitating the targeting of nanoparticles (NPs) in the brain was postulated and investigated by Sun and coworkers [129].

### Solid lipid nanoparticles (SLNs)

Solid lipid nanoparticles (SLNs) commonly have a spherical morphology, characterized by average diameters ranging from 10 to 1000 nm when they are disseminated in an aqueous medium. SLNs are characterized by their ability to solubilize lipophilic compounds due to the presence of a solid lipid core matrix [120]. The lipid core is commonly composed of various substances such as triglycerides (e.g., tristearin), di-glycerides (e.g., glyceryl behenate), monoglycerides (e.g., glycerol monostearate), fatty acids (e.g., stearic acid), steroids (e.g., cholesterol), or waxes (e.g., cetyl palmitate). Its stability is maintained using surfactants, although the utilization of a combination of emulsifiers may offer enhanced effectiveness in preventing the aggregation of particles [130].

**Fig. 10:**
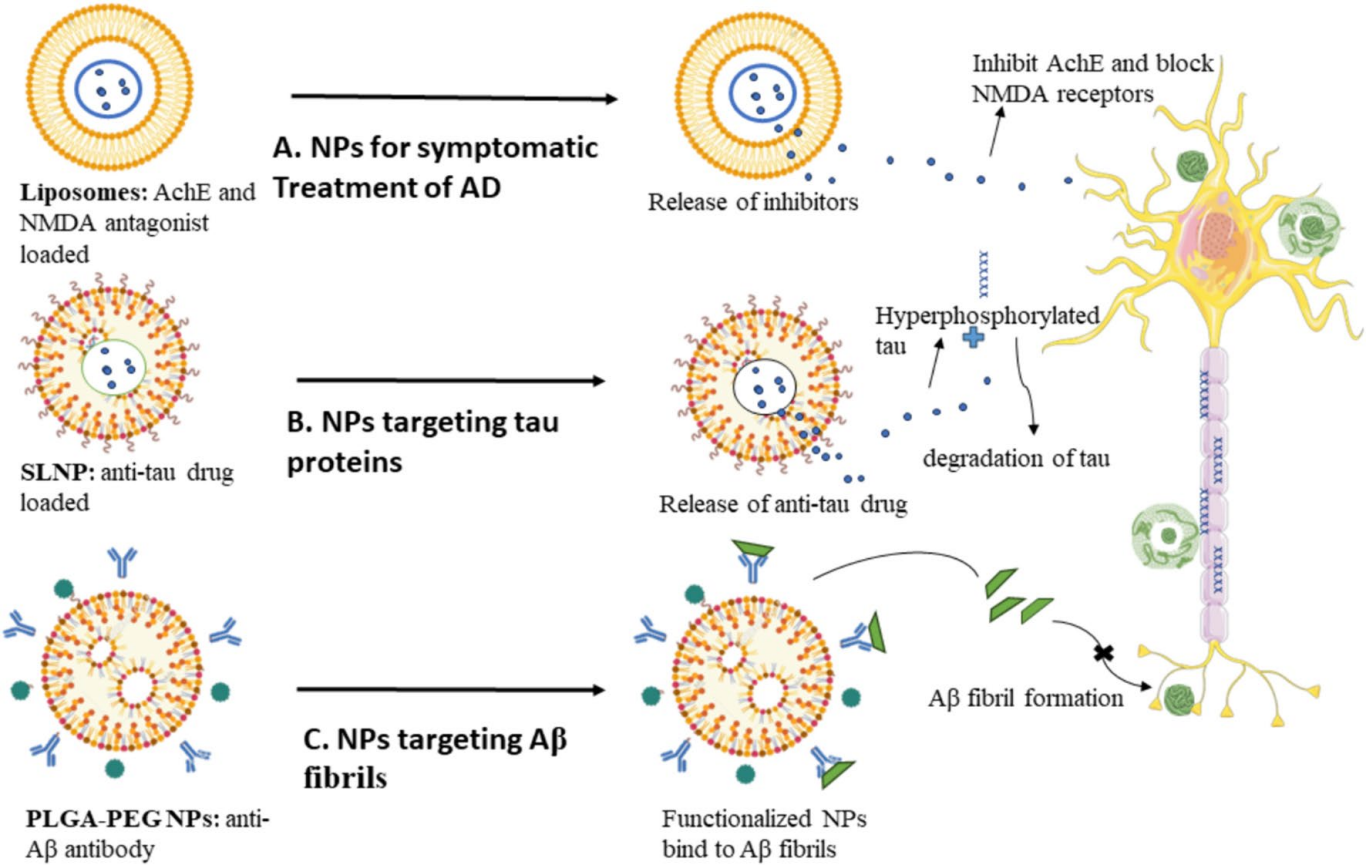
Comparison of effect of NPs in neurons associated with AD after overcoming the BBB **A** Liposome with loaded AchE inhibitors targeting cholinergic system impairment, **B** Functionalized phosphatidylserine in SLNP-loaded anti-tau medication that targets hyperphosphorylated tau Liposomes containing AchE inhibitors that target cholinergic system protein malfunction **C** Anti-Aβ antibody-coated PLGA-PEG is used to target, solubilize, and remove Aβ fibrils

The BBB can be effectively traversed by employing SLNs or nanocarriers composed of lipids. These innovative formulations have demonstrated the ability to facilitate the transport of therapeutic agents to the brain by effectively penetrating the BBB (Fig. 10) or intranasal administration might be employed to circumvent the BBB [131]. Moreover, the utilization of cationic lipids can serve as a viable approach to enhance muco-adhesion within the nasal cavity. This is achieved by facilitating electrostatic interactions with the mucus and the adsorptive-mediated transcytosis of cationic NPs across BBB [132].

## Approaches and challenges

The variety of AD therapy options has expanded thanks to the development of nanotechnology. NPs have surmounted the conventional barriers to medication administration, such as the BBB and the oral/gastric channel barrier [133]. Numerous NPs with proven efficacy guarantee targeted medication delivery at particular pH and temperature levels. Research has demonstrated that numerous characteristics of NPs are involved in their interaction with biological systems, and another study highlights the function of particular metallic nanocarriers in bioaccumulation-mediated neurotoxicity [134]. Nonetheless, certain studies have demonstrated that NPs with a lower size and negative charge can effectively pass the blood–brain barrier and exhibit more inhibitory effects [135]. For AD therapy to be effective, a good biocompatible nanocarrier with the right size, shape, charge, and surface properties relevant to their target specificity is necessary [136]. This will also help with pharmacokinetic and pharmacodynamic profile determination, which will enable precise formulation dose and application route selection. Future research on human clinical trials examining the safety and effectiveness of suitable NPs could lead to the creation of affordable treatments [137].

## Conclusions

The potential of nanotechnology in the development of efficient, secure, and BBB-penetrating nanodrugs for the management of numerous central nervous system disorders deserves acknowledgment. Utilization of nanoparticulate devices to overcome BBB offers significant benefits, which are further augmented by the noninvasive method of drug delivery, enhanced therapeutic outcomes, and diminished occurrence of undesirable consequences. The potential of utilizing receptor-mediated transcytosis for the delivery of pharmaceuticals and diagnostic molecular probes that can cross the BBB has been examined in this context. For example, previous studies have shown evidence that nanocarrier structures coated with PBCA polymer can effectively transport contrast compounds that are impermeable to the blood–brain barrier BBB for neuroimaging.

## Data Availability

Not applicable.
